# Preemptive Therapy for Cryptococcal Meningitis: A Valid Strategy for Latin America?

**DOI:** 10.3390/jof2020014

**Published:** 2016-05-23

**Authors:** José E. Vidal

**Affiliations:** Department of Neurology; Emilio Ribas Institute of Infectious Diseases; Av Doutor Arnaldo 165, Cerqueira César, São Paulo, CEP 05411-000, Brazil; josevibe@gmail.com; Tel./Fax: +55-11-3896-1200

**Keywords:** cryptococcal antigen, cryptococcal meningitis, screening, preemptive therapy

## Abstract

AIDS-related cryptococcal meningitis continues to cause a substantial burden of death in low and middle income countries. Better diagnostics allow detection of cryptococcosis in the asymptomatic phase and using these technologies to screen at-risk persons would likely reduce mortality. The World Health Organization recommends cryptococcal antigen screening among populations with a prevalence of cryptococcal antigenaemia (CRAG) > 3%. There is scarce data about CRAG prevalence in Latin America. Four studies (only one published as a full text) showed asymptomatic CRAG prevalence between 2.7% and 6.2% in several sub-sets of HIV-infected patients. The CRAG lateral flow assay (LFA) has several advantages over other techniques for actual implementation of a screening program. Although more studies are necessary to confirm available data, implementation of the CRAG screening strategy seems to be opportune in Latin America.

## 1. Introduction

*Cryptococcus neoformans* is a major cause of adult meningitis among HIV-infected persons in low and middle income countries. Sub-Saharan Africa has the highest burden [1,2], but Latin America is the region with the third highest prevalence of cases of cryptococcosis [3]. Currently, cryptococcal meningitis represents the main cause of HIV-related opportunistic meningitis in Brazil [4] and in most low- and middle-income countries [5]. Mortality continues to be unacceptable high. In retrospective and prospective hospital-based studies performed in Brazil and Argentina, the case fatality rates have ranged from 26% to 63% [4]. These results are similar to 24%–50% reported in prospective interventional trials in Africa and Asia [6]. These studies suggest that cryptococcosis is an important cause of mortality in Latin America, and that this mortality is potentially preventable.

Key Recommendations to reduce mortality and morbidity due to AIDS-related cryptococcal meningitis has been reviewed elsewhere [4], and early diagnosis of cryptococcal infection is a keystone to improving outcomes. Detectable cryptococcal antigen (CRAG) in peripheral blood precedes meningitis symptoms by weeks to months, offering a relevant opportunity for early detection.

The World Health Organization (WHO) recommended CRAG screening among populations with a prevalence of cryptococcal antigenaemia > 3% [7]. The WHO recommends routine serum or plasma CrAg screening in ART (antiretroviral therapy)-naïve adults a CD4 counts < 100 cells/μL, followed by pre-emptive anti-fungal therapy if CRAG positive, to reduce the development of cryptococcal disease [7].

Thus, to implement WHO recommendations it is necessary to know the local CRAG prevalence in sub-sets of patients.

## 2. Cryptococcal Antigen Prevalence in HIV-Infected Persons from Latin America

The majority of data regarding cryptococcal antigenemia is concerning outpatients in sub-Saharan Africa, where patients with CD4 ≤ 100 cells/μL have a CRAG prevalence reported between 2.2% and 21% with an average of 6.8% (95%CI, 6.5%–7.2%) among studies including only asymptomatic, ART-naïve outpatients [8]. In Southeast Asia, CRAG prevalence in patients with CD4 ≤ 100 cells/μL is reported between 4% and 20.6% or up to 12.9% in studies including only asymptomatic, ART naïve patients [8].

In the WHO recommendations, high prevalence was defined as > 3%, but more recent analyses have reported that screening may be cost-effective even at a prevalence as low as 0.6% [9,10]. Currently, there are no published studies during the ART era of CRAG prevalence in HIV-infected persons from Latin America. There are two unpublished studies regarding CRAG prevalence in the ART era and only one study from the pre-ART era. The first retrospective study was conducted in Lima, Peru among 368 ART-naïve adults with CD4 of ≤ 100 cell/μL without a history of cryptococcosis. In Lima, 3.6% (*n* = 13; 95% CI, 1.7% to 5.5%) were CRAG positive. Three out of these 13 samples developed cerebrospinal fluid culture positive cryptococcosis and they were not considered in the prevalence of isolated CRAG. Thus, 2.7% (10/368) presented with an isolated CRAG-positive [11]. The second was a prospective study conducted in Buenos Aires, Argentina among HIV-infected persons with CD4 ≤ 100 cells/μL, without prior cryptococcosis and without antifungal therapy in the last 14 days. Among 114 patients evaluated, 10 (8.8%; 95%CI, 4.3%–15%) were CRAG positive. Six of these 10 patients presented cryptococcal meningitis. Thus, 3.5% presented isolated CRAG-positive [12]. From the pre-ART era, Negroni reported a 6.2% asymptomatic CRAG prevalence by latex agglutination among 193 HIV-infected persons with CD4 < 300 in Argentina [13].

In the meantime, these results suggest that the implementation of the CRAG screening strategy with preemptive treatment of asymptomatic, early disseminated cryptococcal infection is opportune in Latin America. This approach saves lives and is cost-effective (including the cost savings from preventive therapy). CRAG screening implementation presents individual and public health challenges (for instance, access to health service, provision of fluconazole, lost to follow-up of the patients) [9,14,15]. However, the CRAG lateral flow assay (LFA) has several advantages over CRAG-latex or EIA for actual implementation of a screening program [5,16]. LFA meet many of the World Health Organization Affordable, Sensitive/Specific, User-friendly, Rapid/Robust, Equipment-free, Delivered (WHO ASSURED) criteria for being affordable, sensitive, specific, user-friendly, robust and rapid, equipment-free, and deliverable to those who need it the most.

In conclusion, although more studies are necessary to confirm available data, implementation of the CRAG screening strategy seems to be opportune in Latin America in order to decrease mortality due to HIV-related cryptococcosis.
